# PIEZO1 gain-of-function gene variant is associated with elevated tendon stiffness in humans

**DOI:** 10.1152/japplphysiol.00573.2022

**Published:** 2023-05-25

**Authors:** Tobias Götschi, Victoria Held, Gianna Klucker, Barbara Niederöst, Per Aagaard, Jörg Spörri, Fabian S. Passini, Jess G. Snedeker

**Affiliations:** ^1^Laboratory for Orthopaedic Biomechanics, Department of Orthopaedics, Balgrist University Hospital, University of Zurich, Zürich, Switzerland; ^2^Institute for Biomechanics, ETH Zurich, Zürich, Switzerland; ^3^Sports Medical Research Group, Department of Orthopaedics, Balgrist University Hospital, University of Zurich, Zürich, Switzerland; ^4^Department of Orthopaedics, University Centre for Prevention and Sports Medicine, Balgrist University Hospital, University of Zurich, Zürich, Switzerland; ^5^Department of Sports Science and Clinical Biomechanics, University of Southern Denmark, Odense, Denmark

**Keywords:** jump performance, mechanotransduction, tendon stiffness, ultrasound

## Abstract

Prolonged periods of increased physical demands can elicit anabolic tendon adaptations that increase stiffness and mechanical resilience or conversely can lead to pathological processes that deteriorate tendon structural quality with ensuing pain and potential rupture. Although the mechanisms by which tendon mechanical loads regulate tissue adaptation are largely unknown, the ion channel PIEZO1 has been implicated in tendon mechanotransduction, with human carriers of the PIEZO1 gain-of-function variant E756del displaying improved dynamic vertical jump performance compared with noncarriers. Here, we sought to examine whether increased tendon stiffness in humans could explain this increased performance. We assessed tendon morphological and mechanical properties with ultrasound-based techniques in 77 participants of Middle- and West-African descent, and we measured their vertical jumping performance to assess potential functional consequences in the context of high tendon strain-rate loading. Carrying the E756del gene variant (*n* = 30) was associated with 46.3 ± 68.3% (*P* = 0.002) and 45.6 ± 69.2% (*P* < 0.001) higher patellar tendon stiffness and Young’s modulus compared with noncarrying controls, respectively. Although these tissue level measures strongly corroborate the initial postulate that PIEZO1 plays an integral part in regulating tendon material properties and stiffness in humans, we found no detectable correlation between tendon stiffness and jumping performance in the tested population that comprised individuals of highly diverse physical fitness level, dexterity, and jumping ability.

**NEW & NOTEWORTHY** The E756del gene variant causes overactivity of the mechanosensitive membrane channel PIEZO1 and is suspected to upregulate tendon collagen cross linking. In human carriers of E756del, we found increased patellar tendon stiffness but similar tendon lengths and cross-sectional areas, directly supporting the premise that PIEZO1 regulates human tendon stiffness at the level of tissue material properties.

## Graphical abstract

**Figure:**
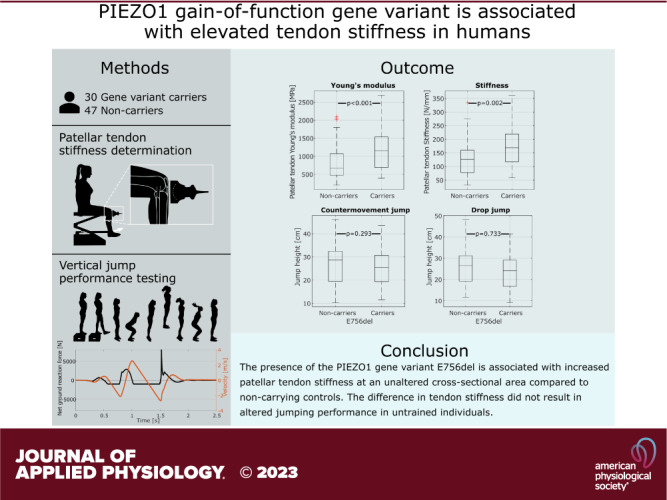

## INTRODUCTION

Tendon tensile mechanical properties are of key importance for human motion and sports performance. During jumping, the patellar tendon is required to bear forces up to six times body weight (1). The capability of tendons to store and subsequently release strain energy increases movement efficiency (2, 3) and prevents excessive heat accumulation (4, 5). Repetitive exertion of tensile stress can promote remodeling leading to an increase in tendon stiffness (6–12), failure strength (13), and efficiency of force transmission (8, 14). These adaptations are the result of structural or compositional remodeling processes, such as enzymatic collagen cross linking and tendon hypertrophy (15–17). Paradoxically, repetitive high loading constitutes a main risk factor for tendinopathy, a degenerative process comprising fibrotic changes with tenocyte proliferation and neovascularization, manifesting in pain and reduced tendon strength (18–21). Although the pathways whereby tendon-resident cells sense extrinsic mechanical loads remain poorly understood, we recently identified PIEZO1 as a crucial molecular force sensor in tendon cells (22). PIEZO1 is a known mechanosensitive ion channel that is involved in various physiological processes (23, 24), including bone formation (25). We found that stretching of tail tendon fascicles from wild-type mice induces a transient Ca^2+^ influx into tenocytes, whereas PIEZO1 knockout mouse tenocytes display a reduced reaction to the same stimulus, indicating that PIEZO1 is involved in converting mechanical forces into biological signals (22). Accordingly, PIEZO1 gain-of-function (GOF) mice develop stiffer and stronger flexor tendons than wild-type mice, likely caused by PIEZO1 overactivity and subsequently elevated tendon remodeling processes.

PIEZO1 GOF alleles also exist in humans. Among them, the E756del variant causes a prolonged activation time of the ion channel upon a mechanical stimulus, leading to a stronger Ca^2+^ influx (26, 27). E756del has been associated with malaria resistance, which is suggested to constitute a selective advantage within regions of high malaria prevalence, resulting in PIEZO1 GOF being present in one-third of the African population (26). In our recent study based on a cohort of American college students, we found that E756del carriers demonstrate a higher drop jump to countermovement jump height ratio than noncarrier controls, indicating that PIEZO1 also has functional relevance in human tendons (22). Over-representation of the gene has since been confirmed in elite sprinters (28). Interestingly, these two studies speculated that different mechanisms are at work with the study of Passini et al. (22) speculating that increased tendon stiffness, and increased rate of muscle force transmission to the skeleton, is responsible for the observed performance gain, whereas Nakamichi et al. (28) speculate that lower tendon stiffness but increased energy storage capacity underlies the observed performance advantages. In any case, whether and how PIEZO1 regulates tendon mechanical properties in humans remains unknown. Adaptive stiffness regulation is a core process in tendon physiology, and identifying respective molecular targets represents a key step toward the development of novel therapeutic strategies.

In this study, we tested whether PIEZO1 is a stiffness regulator of human tendons by assessing mechanical properties, morphology, and functional performance of human lower limb tendons in E75del carriers compared with noncarrier controls.

## MATERIALS AND METHODS

### Study Design

We recruited 77 adults of self-reported Middle and West African descent residents in the vicinity of Zurich Switzerland. Exclusion criteria were inability to perform vertical jump testing and medical conditions or comorbidities such as manifesting signs of tendon pathologies, injury to the lower extremity, and systemic pathologies relevant to the purpose of the study. All participants received detailed written information about the measurement procedures and the purpose of the study and provided their informed written consent. The project was reviewed and approved by the local ethics committee (BASEC 2021-00198). The study was powered based on the jump performance data published by Passini et al. (22), indicating 77 subjects to be required to reach a power of 90% for detecting a difference between E756del carriers compared with noncarriers provided the proportion of the former reaches a minimum of 1/4 in the study population.

### Questionnaires

To rule out the presence of lower limb tendinopathy, the Victorian Institute for Sports Assessment questionnaires for the Achilles (VISA-A) and the patellar tendon (VISA-P) were administered (29–32). Further questionnaires were used to assess the self-reported physical activity profile of each participant, including the six-level physical activity scale (PAS), which takes into account both the duration and intensity of physical activity in various domains, and the Tegner Activity Scale (TAS), covering activities of daily life as well as participation in specific recreational and competitive sports (33, 34). We acquired information on physical activity specifically pertaining to sport disciplines with high body accelerations using the Marx questionnaire, addressing both the most active state in the last year as well as for the last 10 years (35). For all of the acquired activity scales, higher scores represent higher activity levels.

### Morphological Assessments

Morphological assessments were performed using ultrasound (US) brightness mode imaging (Aixplorer Ultimate, SuperSonic imagine, Aix-en-Provence, France) with a linear transducer (SL18-5). The Achilles tendon (AT) was examined with the subject lying prone on the examination bed with the foot hanging over the edge. The tendon cross-sectional area (CSA) was measured 2 cm proximal to the calcaneal insertion. In a subset of the study population (the last 39 subjects in the order of appearance for the study visit), we additionally measured AT length (defined as the distance between the calcaneal insertion and the most distal part of the gastrocnemius musculotendinous junction), as well as medial and lateral gastrocnemius muscle pennation angles, which were averaged before further processing. For patella tendon (PT) assessments, the subject sat upright with adjustable support underneath the knee with a knee flexion angle of ∼20°. PT CSA was measured 1 cm distal to the patella apex, and its length was defined as the distance between the patellar and tibial insertion. The morphological assessment and patellar tendon tensile property measurements described in the paragraph below were conducted on the dominant leg, which was defined as the leg; the subject would reportedly use for kicking a ball (36).

### Assessment of Patellar Tendon Tensile Properties

**Figure 1.**
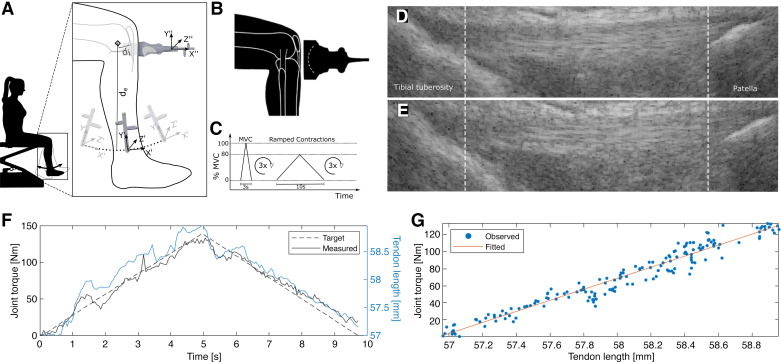
Schematic of the measurement procedure used to determine the tensile properties of the patellar tendons. *A*: the subject performed knee flexion-extension, during which the shank trajectory was recorded with an optical tracking system to locate the instantaneous axis of knee rotation. Subsequently, an optically tracked ultrasound transducer was used to determine the extensor apparatus’s internal moment arm (d_i_). d_e_: external moment arm. *B*: patellar tendon elongation during isometric contraction was tracked with a convex ultrasound transducer. *C*: voluntary contraction profile used in the study. *D*: exemplary ultrasound tendon length measurement during voluntary contraction at low and high tendon stress (*E*). *F*: linear ramped contraction torque superimposed with the corresponding tendon length measurement. *G*: tendon length with respect to the produced joint torque was fitted with a linear regression to estimate tendon stiffness. MVC, maximum voluntary contraction.

Before the assessment, the participant completed a 5-min warm-up on a stationary bike at self-selected speed. The participant was then seated on the examination bed with the lower legs hanging over the edge. To determine the internal moment arm of the patellar tendon and thereby derive tendon force given an external torque, the trajectory of the lower limb during small flexion extension movements (±10°) at ∼90° of knee flexion was recorded using optical markers and an infrared tracking system [FusionTrack 500, Atracsys LLC, 7 Hz sampling frequency, tracking accuracy 0.09 mm (RMS)], immediately followed by the determination of the position of the patellar tendon by means of a marker-equipped ultrasound transducer (Fig. 1*A*). The axis with minimum distance variation to any point of the lower limb trajectory represents the instantaneous axis of knee rotation, and its orthogonal distance to the patellar tendon yields the internal moment arm. The participant’s ankle was then connected to a force sensor (FS06-200 kg, Forsentek Co., Limited, PR China) at a fixed knee flexion angle of 90°, and the distance between the ankle strap and the lateral knee epicondyle was measured to determine the external moment arm. The participant was instructed to hold on to the handrails and to remain seated upright throughout the procedure. To allow well-controlled force application, the participant was presented with a visual representation of the target force profile and the currently applied force. Initial force profiles were designed to get the participant accustomed to the system. Then, three maximum voluntary isometric contractions (MVICs) were measured. Subsequently, a linear symmetric ramp contraction with a duration of 10 s reaching 80% maximum force was conducted, during which tendon elongation was recorded. Patellar tendon elongation was recorded (sampling rate: 18 Hz) with the US device used for morphological assessment connected to a curvilinear transducer array (XC 6-1) for a larger lateral field of view. To enable acoustic coupling between the convex US array and the skin, the probe was equipped with a custom-made standoff pad casted from a gelatinous material (Formaform, Glorex AG, Switzerland) (Fig. 1*B*). The measurement was repeated until three trials of sufficient quality were recorded (Fig. 1*C*). For the analysis of the acquired data, with a semiautomatic script, tendon length throughout the contraction was determined by tracking the patellar and the tibial insertion of the patellar tendon frame by frame (Fig. 1, *D–F*). Between 20% and 80% MVIC, tendon elongation was assumed to respond linearly to the applied stress, and consequently, tendon stiffness was derived by applying a linear fit over both the eccentric and the concentric phase of tendon loading in this stress region (Fig. 1*G*). Assuming tensile stress to be uniformly distributed over the tendon’s CSA and the latter to be uniform throughout its length, the tendon tensile Young’s modulus was estimated by normalizing stiffness by both the initial tendon length and CSA.

### Vertical Jump Performance Testing

**Figure 2.**
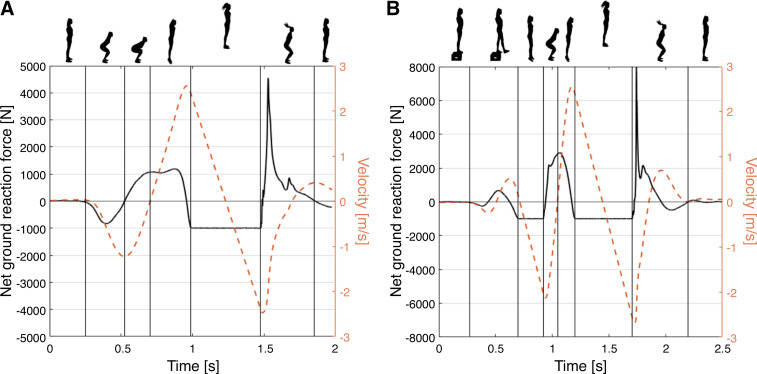
Exemplary ground reaction force and derived center of mass vertical velocity during a countermovement jump (*A*) and a drop jump (*B*).

The jump protocol and the derived parameters were intended to provide indicators of tendon mechanical behavior in various domains. Standing on a force plate (9260AA, Kistler AG, Switzerland), the participants performed maximal vertical countermovement jumps (CMJs) and drop jumps (DJs), respectively, both legged and on either single leg. For standardization purposes, the participants performed the test with bare feet and were instructed to place their hands on the hip throughout the entire maneuver. For the CMJ, the participants started in an upright position, squatted down to a self-selected depth, thrusted themselves up to jump as high as possible, and ended the task in a stable upright position upon landing (Fig. 2*A*). The DJ was initiated with the participants standing on a 20 cm box also instrumented with a force plate, stepping down onto the force plate and immediately upon ground contact initiated the thrusting phase, again completing the task in a stable upright position after the second ground contact (Fig. 2*B*). During instruction for the drop jump, emphasis was given on minimizing the ground contact time after the drop. The participants were allowed ample practice time to become accustomed to each exercise. Before and during execution, the participants were verbally encouraged to exert maximal effort. A minimum of three repetitions of each jump variant were recorded.

Jump parameters were determined based on the vertical component (Fz) of the ground reaction force signal recorded by the force plates (sampling rate: 1 kHz). Jump height was computed based on the time in air and was intended to provide a proxy for the functional capacity of the tendons involved in the posterior chain. The jump height ratio, the ratio between DJ and CMJ height, represented tendon functional efficiency during high stress stretch shortening. The maximum instantaneous power during the propulsive phase of either jump type was computed to yield an estimate of the elastic recoil capabilities of the muscle-tendon complex. The ground contact time during the DJ was chosen as a proxy for the tendon high strain rate stiffness. Table 1 provides information on the assessed jump parameters, including the repetition aggregation applied.

**Table 1.** Jump parameters used in the study

Parameter	Calculation	Tendon Characteristics	Repetition Aggregation	Unit	Jump Variant
Jump height	Time from takeoff to landing	Functional capacity	Max	cm	CMJ, DJ
Jump height ratio	Jump height ratio between DJ and CMJ	Efficiency of high-stress stretch-shortening cycle	N/A		CMJ, DJ
Maximum propulsive power	Maximum instantaneous power during the propulsive phase of the DJ	Elastic recoil	Max	W	CMJ, DJ
Ground contact time	Time from landing to takeoff (last ground contact)	High strain rate stiffness	Min	s	DJ

CMJ, countermovement jump; DJ, drop jump; N/A, not applicable.

### Genotyping for the PIEZO1 Gain-of-Function E756del Variant

At the end of the study visit, saliva samples were collected with a DNA collection kit (OG-500, DNA Genotek, Canada). Genomic DNA was isolated according to the manufacturer’s instructions (prepIT-L2P, DNA Genotek, Canada). The E756 locus was amplified with the primers described in Ma et al. (26), and the amplicon was sequenced (Microsynth AG, Switzerland) to distinguish E756del carriers from noncarriers.

### Statistical Analysis

We used a multivariate approach to estimate the effect of PIEZO1 GOF E756del on the outcomes of interest while controlling for other potentially confounding variables. Because including all factors into one model can lead to overfitting and yield erroneous estimates, we determined the linear model for inference by selecting the set of predictors with the best predictive performance in a leave-one-out validation scheme (maximum adjusted *R*^2^). We constrained the optimization procedure in that the factor encoding the presence or absence of the allele variant of interest was present in every iterated model. As a consequence of this approach, no effect estimates are reported (or available) for factors not included in the final models. A list of all factors included in the different outcome domains is provided in Supplemental Table S1 (https://doi.org/10.6084/m9.figshare.22440172.v1). Higher-order polynomials (with interaction effects) and alternative transformations were also evaluated but were deemed unsuitable in the current context (data not shown). Statistical significance was set at α = 0.05. The analysis was conducted in R (37) and MATLAB (2020a, The MathWorks, Inc., Natick, MA).

## RESULTS

### Participant Characteristics

To test whether PIEZO1 regulates human tendon mechanical properties, we recruited 77 participants from a population with high E756del frequency. Of these 77 participants, we identified 30 (39%) E756del carriers, of whom three were homozygous. Females were identically distributed between both groups, accounting for a proportion of 40% each. However, E756del carriers were older and less physically active on average than the noncarrier group. The two groups were comparable in average body height, but the E756del carrier group was 7.2 kg heavier on average (Table 2).

**Table 2.** Participant characteristics of PIEZO1 E756del carriers and noncarriers

Demographic Data	E756del Carriers (*n* = 30 | 39%)	Noncarriers (*n* = 47 | 61%)
Female	12 (40%)	19 (40%)
Age, yr	36.0 ± 12.9	30.1 ± 8.57
Height, cm	175 ± 9.25	173 ± 9.51
Weight, kg	84.2 ± 19.7	77.0 ± 15.0
BMI, kg·m^−2^	27.2 ± 5.27	25.8 ± 4.21
Right-footed	28 (93%)	42 (89%)
PAS [1–6]	4.03 ± 1.56	4.34 ± 1.45
Tegner [0–10]	4.50 ± 2.01	4.66 ± 1.56
Marx 1 year [0–16]	5.67 ± 5.16	8.36 ± 4.94
Marx 10 year [0–16]	9.60 ± 5.72	12.0 ± 4.34

Data are presented as the means ± SD or counts and percentages. PAS, physical activity scale.

### Tendon Morphology

Descriptive statistics of the two study groups indicated tendon morphology to be comparable (Table 3). Indeed, the linear models used for inference revealed no difference between the groups.

**Table 3.** Descriptive statistics of tendon morphology among the two study groups

Parameter	Tendon	E756del Carriers (*n* = 30)	Noncarriers (*n* = 47)
Cross-sectional area, mm^2^	AT	71.1 ± 12	71.3 ± 11.4
	PT	97.7 ± 14	96.8 ± 16
Length, mm	AT	22 ± 2.6	21.1 ± 2.8
	PT	50.6 ± 4.7	49.7 ± 5.5

Data are presented as the means ± SD. Note: at length was assessed on 39 individuals only. AT, Achilles tendon; PT, patellar tendon.

Achilles and patellar tendon length and CSA were associated with the subject height, and CSA was in addition associated with the subject’s body-mass index (BMI) (Table 4).

**Table 4.** Inference models for tendon morphological characteristics

Outcome	Tendon	E756del	Male Sex	Age, yr	Height, m	BMI, kg·m^−2^	Adjusted *R*^2^
Cross-sectional area, mm^2^	AT	−3.06 (0.177)			62.6 (<0.001)	0.723 (0.003)	0.342
	PT	−2.3 (0.414)	7.12 (0.071)		65.8 (0.002)	0.832 (0.005)	0.416
Length, mm	AT	0.427 (0.502)			20.5 (<0.001)		0.563
	PT	0.889 (0.467)		−0.0862 (0.121)	19.3 (0.003)		0.106

Linear models with maximum cross-validated performance for tendon morphological parameters. The factors included in each inference model are described with their respective coefficients and *P* values based on a linear regression (in brackets). Note: the factor encoding the presence or absence of the PIEZO1 gain-of-function allele was included in all evaluated models irrespective of its predictive value. AT, Achilles tendon; PT, patellar tendon.

### Tendon Tensile Properties

**Figure 3.**
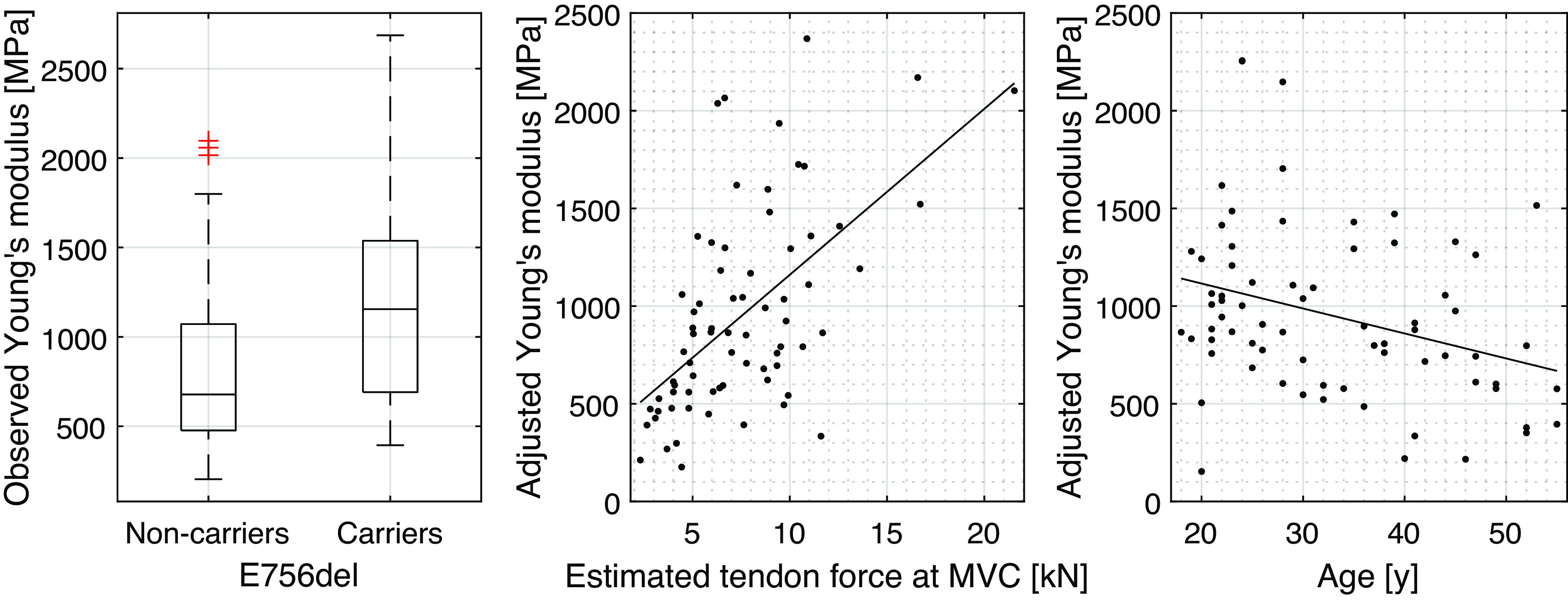
Patellar tendon Young’s modulus as a function of the two investigated groups, estimated tendon force at maximum voluntary contraction (MVC) and subject age. In the scatterplots the response variable is adjusted for the influence of the predictors not included in the respective plot as estimated based on the linear inference model presented in Table 5. Tendon-force and age-associated fits are represented with solid lines.

There was a large overall variability in PT stiffness and Young’s modulus [coefficient of variation (SD/mean): 0.587 and 0.597, respectively]. Nevertheless, E756del carriers exhibited significantly higher tendon stiffness (2,279 ± 1,250 vs. 1,558 ± 861 N·mm^−1^, *P* = 0.002) and Young’s modulus (1,194 ± 617 vs. 820 ± 498 MPa, *P* < 0.001) compared with noncarriers (Fig. 3). Table 5 lists the factors that were identified to be predictive for the assessed tendon tensile properties and the related coefficients and *P* values. The estimated tendon force produced by the subject during MVC was found to be highly predictive of PT stiffness and Young’s modulus. Additional descriptive statistics of factors related to PT tensile measurements are provided in the Supplemental Material (Supplemental Table S2; see https://doi.org/10.6084/m9.figshare.22440172.v1).

**Table 5.** Inference models for tendon tensile properties

Outcome	E756del	Estimated Tendon Force at MVC, N	Age, yr	External Moment Arm, cm	Adjusted *R*^2^
Stiffness, N·mm^−1^	669 (0.002)	0.165 (<0.001)			0.390
Young’s modulus, MPa	485 (<0.001)	0.0902 (<0.001)	−12.0 (0.023)	−43.7 (0.060)	0.422

Linear models with maximum cross-validated performance for tendon morphological parameters. The factors included in each inference model are described with their respective coefficients and *P* values based on a linear regression (in brackets). Note: the factor encoding the presence or absence of the PIEZO1 gain-of-function allele was included in all evaluated models irrespective of its predictive value.

### Vertical Jump Performance

None of the assessed vertical jump performance measures indicated a significant difference between E756del carriers and noncarriers. Outcome parameters were associated with various confounders, with adjusted *R*^2^-values reaching 0.766 in one instant. In particular, subject age, sex, and body weight-normalized knee extensor strength consistently yielded high predictive value for jump performance. Inference models for the nondominant leg jumps are summarized in Table 6. Descriptive statistics and bilateral and dominant leg vertical jump inference models are presented in the Supplemental Material (Supplemental Tables S3 and S4, respectively; see https://doi.org/10.6084/m9.figshare.22440172.v1).

**Table 6.** Inference models for the vertical jump performance of the nondominant leg of the subjects

Outcome	Jump Type	E756del	Male Sex	Age, yr	BMI, kg·m^−2^	Body Weight, kg	Maximum Torque Per Body Weight, Nm/kg	Marx Score 1 Year	Physical Activity Score	Gastrocnemius Pennation Angle [°]	Achilles Tendon Length, cm	Patellar Tendon Cross-Sectional Area, mm^2^	Adjusted *R*^2^
Jump height, cm	CMJ	0.701 (0.293)	4.55 (<0.001)	−0.127 (<0.001)		−0.0768 (0.001)	0.939 (0.059)		0.84 (<0.001)				0.693
	DJ	0.282 (0.733)	4.61 (<0.001)	−0.104 (0.027)	−0.291 (0.006)		1.37 (0.031)	0.201 (0.039)					0.640
Jump height ratio	Both	0.0047 (0.928)									0.0325 (0.013)		0.065
Maximum propulsive power, W	CMJ	74.7 (0.33)	601 (<0.001)	−14.7 (<0.001)	52 (<0.001)			25.7 (0.004)				7.2 (0.019)	0.722
	DJ	−77.1 (0.471)	368 (0.013)	−21.9 (<0.001)		16.8 (<0.001)		50.7 (<0.001)		−38.8 (0.078)		6.5 (0.146)	0.664
Ground contact time, s	DJ	−7.9e-3 (0.505)						−3.4e-3 (0.004)					0.096

Linear models with maximum cross-validated performance for tendon morphological parameters. The factors included in each inference model are described with their respective coefficients and *P* values based on a linear regression (in brackets). Note: the factor encoding the presence or absence of the PIEZO1 gain-of-function allele was included in all evaluated models irrespective of its predictive value. CMJ, countermovement jump; DJ, drop jump.

## DISCUSSION

This study corroborates the role of PIEZO1 as a major regulator of tendon mechanical properties in humans. Carriers of the PIEZO1 Edel756 gain of function (GOF) allele displayed nearly 50% higher patellar tendon stiffness and Young’s modulus than noncarriers. In line with previous investigations, the observed elevation in tendon stiffness seems to be primarily a result of structural or compositional factors rather than tendon cross-sectional area, as no overt differences in tendon morphology were observed. Given that our study included only three homozygous carriers, we do not have sufficient data about the role of the carrier’s zygosity in this regard.

Whereas PIEZO1 GOF carriers showed an increased DJ/CMJ height ratio compared with noncarriers in our previous investigation (22), the current cohort, comprising a more athletically diverse population than our previous study, revealed no detectable difference in vertical jump performance. Jump performance outcome parameters were designed to provide proxies for different aspects of tendon mechanical function. During both the CMJ and DJ, a descending (“eccentric”) phase introduces preloading and elongation (strain) to the muscle-tendon complex. In the muscle, eccentric stretching may increase its concentric force (and hence impulse) potential due to utilization of an intrinsic stretch reflex (38) as well as force potentiation (39, 40), although this theory has been contested (41). A portion of the kinetic energy is converted into tendon strain energy, of which a part is released during the propulsion phase. By dropping down from an elevated position (such as during DJ), the available kinetic energy is increased, accentuating this tendon stretch-release mechanism. By calculating the ratio of DJ height relative to CMJ height, we attempted to amplify the tendon-related component of the observed physical performance (42). Executing a DJ successfully and thereby utilizing passive tendon energy storage requires coordination and sufficient muscular strength (43, 44). Athletes who are proficient in this task achieve ground contact times on the order of 250 ms accompanied by large vertical ground reaction (Fz) forces and consequentially induce very high tendon stress (43). Our study population, however, included only a few subjects who were trained in such plyometric movements, which is confirmed by considerably longer ground contact times on average (bilateral: 327 ± 67 ms). Hence, it is possible that a considerable portion of our participants would be unable to fully utilize increased tendon stiffness as a performance-enhancing mechanism, which would thus prevent the identification of the tendon phenotype influencing jumping performance. Future investigations may therefore narrow the inclusion criteria to construct a cohort composed of participants that are more likely to be physically capable of and well accustomed to the required task.

Notably, however, the jump height ratio was associated with Achilles tendon length. A longer tendon may both increase the capacity for tendon energy storage and decrease the muscular work required for successful completion of the maneuver. Another potentially valuable parameter for assessing physical performance-relevant tendon phenotypes could be the maximum ground reaction force achieved during a plyometric jump. Provided the subject does not establish contact with the heel during deceleration, the magnitude of vertical ground contact force (Fz) in combination with relatively short contact times (compared with CMJ) would likely cause the DJ to be associated with rapid high-strain tendon elongation that in turn will be affected by tendon stiffness, which thus represents a pivotal parameter for explosive motions, such as sprinting (45). In the majority of our subjects, however, limitations in muscular strength rendered heel ground contact inevitable. In pursuit of a related parameter, we analyzed the maximum instantaneous power achieved during the propulsive phase of the DJ and the CMJ.

Our finding of a negative association of subject age with patellar tendon Young’s modulus is in line with and adds the existing body of knowledge on this topic (46). Regarding the presence of the strong positive relationship between the tendon Young’s modulus and the estimated tendon force at the MVC, two underlying mechanisms are conceivable. First, tendon mechanical properties are likely correlated with the subject’s maximal knee extensor strength (8, 47). Second, the observed association may be the result of the erroneous assumption of a linear force-elongation behavior as observed on US. Irrespective of the underlying cause, the observed relationship stresses the importance of performing adequate confounder adjustments.

There are limitations to this study that need to be considered. Logistical constraints prevented us from acquiring ground-truth measurements of PT moment arm length, such as those available with flexed-knee magnetic resonance imaging (7, 48). The validity of estimating PT moment arm length based on simple anthropometric measurements (47, 49) has been questioned (48). Hence, we opted for the approach of combining motion-tracked knee flexion with US imaging. Our measurements yielded shorter moment arms on average compared with the literature-reported values [35 ± 9 mm vs. 42 ± 4 mm, respectively (48)], which may have limited the quantitative validity of our tendon mechanical properties but is unlikely to have resulted in systematic between-group effects. Coactivation of knee flexion muscles during ramped contraction may potentially bias the inferred tendon force (50). Some investigators utilize electromyographic measurements and assume a linear muscular activity-torque relationship to correct for antagonist-induced reductions in net torque. On the other hand, as has been pointed out previously, the error originating from such an estimation may be larger than when considering the produced torque alone, and we, therefore, did not include such data correction (51). These and potentially unidentified error-inducing processes apparently yielded a variability in observed tendon tensile properties, which is likely larger than the true population variability although tendon stiffness has been reported to vary by a factor of ten in some instances (6).

Finally, from a functional perspective, in addition to the present data derived on tendon stiffness, information regarding the implications of PIEZO1 GOF on the viscoelastic tendon properties would be desirable. Such properties could be probed by inspecting the hysteresis behavior of the tendon force over a tendon loading–unloading cycle. Although such in vivo analyses have been published previously (14, 52, 53), technical and human neuromuscular limitations could invalidate measurements (5, 51, 54), and we decided to refrain from conducting this analysis.

### Conclusions

PIEZO1 GOF carriers display substantially elevated patellar tendon stiffness at an equal cross-sectional area compared with noncarriers. In untrained individuals, the difference in tendon stiffness does not translate to detectable differences in jumping performance. Our findings corroborate the premise that the PIEZO1 membrane channel is a significant factor in regulating structural and/or compositional homeostasis in human tendons and that the E756del PIEZO1 mutation is associated with an increased elastic modulus of the load-bearing patellar tendon.

## DATA AVAILABILITY

Data will be made available upon reasonable request.

## SUPPLEMENTAL DATA

10.6084/m9.figshare.22440172.v1 Supplemental Tables S1–S4: https://doi.org/10.6084/m9.figshare.22440172.v1.

## GRANTS

The authors received no specific financial support for the research, authorship, and/or publication of this article.

## DISCLOSURES

No conflicts of interest, financial or otherwise, are declared by the authors.

## AUTHOR CONTRIBUTIONS

T.G., V.H., G.K., B.N., P.A., J.S., F.S.P., and J.G.S. conceived and designed research; T.G., V.H., G.K., B.N., and F.S.P. performed experiments; T.G., V.H., G.K., B.N., and F.S.P. analyzed data; T.G., V.H., G.K., P.A., J.S., F.S.P., and J.G.S. interpreted results of experiments; T.G. and V.H. prepared figures; T.G. and V.H. drafted manuscript; T.G., P.A., J.S., F.S.P., and J.G.S. edited and revised manuscript; T.G., V.H., G.K., B.N., P.A., J.S., F.S.P., and J.G.S. approved final version of manuscript.
